# Evaluation of microglia activation related markers following a clinical course of TBS: A non-human primate study

**DOI:** 10.1371/journal.pone.0301118

**Published:** 2024-05-16

**Authors:** Lucero Aceves-Serrano, Jason L. Neva, Jonathan Munro, Irene M. Vavasour, Martin Parent, Lara A. Boyd, Doris J. Doudet

**Affiliations:** 1 Department of Medicine, Division of Neurology, University of British Columbia, Vancouver, British Columbia, Canada; 2 Faculté de Médecine, École de Kinésiologie et des Sciences de l’activité Physique, Université de Montréal, Montreal, Quebec, Canada; 3 Centre de Recherche de l’institut Universitaire de Gériatrie de Montréal, Montreal, QC, Canada; 4 CERVO Brain Research Centre, Laval University, Quebec City, Quebec, Canada; 5 Faculty of Medicine, UBC MRI Research Center, University of British Columbia, Vancouver, British Columbia, Canada; 6 Faculty of Medicine, Department of Physical Therapy, University of British Columbia, Vancouver, British Columbia, Canada; 7 Faculty of Medicine, Graduate Program of Rehabilitation Sciences, University of British Columbia, Vancouver, British Columbia, Canada; Harvard Medical School, UNITED STATES

## Abstract

While the applicability and popularity of theta burst stimulation (TBS) paradigms remain, current knowledge of their neurobiological effects is still limited, especially with respect to their impact on glial cells and neuroinflammatory processes. We used a multimodal imaging approach to assess the effects of a clinical course of TBS on markers for microglia activation and tissue injury as an indirect assessment of neuroinflammatory processes. Healthy non-human primates received continuous TBS (cTBS), intermittent TBS (iTBS), or sham stimulation over the motor cortex at 90% of resting motor threshold. Stimulation was delivered to the awake subjects 5 times a week for 3–4 weeks. Translocator protein (TSPO) expression was evaluated using Positron Emission Tomography and [^11^C]PBR28, and myo-inositol (mI) and N-acetyl-aspartate (NAA) concentrations were assessed with Magnetic Resonance Spectroscopy. Animals were then euthanized, and immunofluorescence staining was performed using antibodies against TSPO. Paired t-tests showed no significant changes in [^11^C]PBR28 measurements after stimulation. Similarly, no significant changes in mI and NAA concentrations were found. Post-mortem TSPO evaluation showed comparable mean immunofluorescence intensity after active TBS and sham delivery. The current study suggests that in healthy brains a clinical course of TBS, as evaluated with in-vivo imaging techniques (PET and MRS), did not measurably modulate the expression of glia related markers and metabolite associated with neural viability.

## Introduction

Repetitive transcranial magnetic stimulation (rTMS) has been evaluated as a therapeutic intervention for the treatment of various neuropsychiatric disorders. The beneficial effects of rTMS in different conditions are increasingly recognized [1] but its neurobiological basis are still being investigated. Existing studies exploring the neuronal impacts of stimulation have demonstrated its ability to induce alterations in neurotransmission [2,3], long-term plasticity [4], and cell survival [5]. While the potential of repetitive TMS to modulate activity in a diverse range of cells is acknowledged, there remains a notable gap in our understanding, particularly in its effects on microglia.

Microglia are the primary resident immune cells of the central nervous system yet limited research has systematically examined the influence of rTMS on these pivotal cellular elements within the brain. Microglia are one of the key cellular mediators of the neuroinflammatory process, taking part in immune response, tissue repair, plasticity, and spontaneous neural network rewiring [6,7]. These cells can also respond to electrical activity involved in neuronal signaling [8], making it a potential mediator of rTMS effects [9].

Given microglia’s role in immune activation in response to injury [7], evaluating their reactivity could also help elucidate the impact of rTMS on neuroinflammation processes and potential injurious effects. Reports on rTMS safety have mainly assessed patient comfort (i.e., headache, neck pain, hearing, and others) and seizure risk, with limited studies examining molecular and cellular markers [10,11]. As new rTMS paradigms with a higher number of pulses, frequency, and intensity are developed, assessment of safety is imperative, especially in interventions of prolonged exposure. The assessment of neuroinflammatory markers is relevant in evaluating rTMS safety as studies have shown the long-term detrimental effect of even low-grade inflammation, as observed in neurodegenerative disorders [12–17].

To our knowledge, very few studies have investigated glia response to chronic rTMS in the context of safety [10,11]. An early evaluation by Liebetanz *et al*. assessed glial activity after five days of 1Hz rTMS delivery in rats [11]. They reported no changes in astrocyte or microglia activation and proliferation. Similarly, a study of very high frequency (100 Hz) rTMS found no microglia nor astrocyte reactivity at the stimulated site after three stimulation sessions [10]. While these studies are relevant, their comparability to human clinical studies is limited due to the nature of the animals used (e.g., small head and brain and neuroanatomical differences), the delivery of very high frequency rTMS, and the small number of stimulation sessions.

Evaluation of glial activation in vivo can be carried using tools such as positron emission tomography (PET), which has proven to be a valuable tool for assessing neuroinflammatory processes by targeting the translocator protein (TSPO) [18,19], a molecule that is over-expressed in activated microglia [20]. PET-TSPO studies in animals have shown an increase in TSPO expression following neuroinflammation induction [21,22], while studies in humans have reported upregulation of TSPO after stroke and traumatic brain injury near the lesion site [23–26]. Magnetic resonance spectroscopy (MRS) also allows in vivo evaluation of metabolites associated with neuroinflammatory processes and neural integrity [27]. MRS measurements of *myo*-inositol (mI), an osmolyte that is particularly prominent in astrocytes [28,29], have been used as indirect markers of neuroinflammatory process. Concentrations of this metabolite are elevated in the presence of neuroinflammation as reported in individuals with multiple sclerosis [30], traumatic brain injury [31] and neurodegenerative disorders [32]. MRS can also be used to evaluate N-acetyl-aspartate (NAA) concentration, a neuronal and myelin marker as it is localized within neurons and oligodendrocytes [33]. A decrease in NAA is thought to indicate cellular dysfunction and damage and underlying dysfunction in myelin maintenance [33–35]. In studies of traumatic brain injury and stroke, NAA levels are reduced proportionately to the degree of tissue damage [36–38] and remain depressed for hours to weeks, depending on injury severity [37,39,40].

In the current work, we took advantage of PET and MRS to assess the effects of a clinical course of rTMS on activating inflammatory markers. We also carried out post-mortem immunofluorescence imaging to evaluate changes in TSPO expression. The study was carried in macaque monkeys for their comparable neuroanatomy to humans and larger brains compared to other animal species. Specifically, we evaluated the effects of theta burst stimulation (TBS)–a patterned high frequency (50Hz) rTMS paradigm–when delivered daily for three to four weeks over the left motor cortex (M1).

## Materials and methods

### Animals

All non-human primates (NHP) experiments were conducted ethically following the guidelines and regulations from the Canadian federal government on animal welfare and were approved by the Committee on Animal Care at the University of British Columbia. A total of eleven healthy rhesus macaque monkeys were used (*Macaca mulatta*; 4 females and 7 males; age 9.3 ± 4 years; weight 7.6 ± 1.7 kg). The animals were housed in pairs or groups in large pens with indoor-outdoor access and kept on a 12-hour light cycle. The Animal Research Unit staff monitored the animals twice daily for health and behavior. Observations regarding alertness, interactions with pen mate, appetite, willingness to come for treats and evidence of defecation were monitored. Animals received regular chow, fresh fruits and vegetables, and a variety of nuts scattered inside their cage to encourage foraging. Enrichment was provided in the form of toys as well as water tubs to play during the summer months. Animals interacted constantly with research and animal care staff. Prior to the start of the study, animals were trained to the pole and collar method [41,42].

NHPs were randomly assigned to receive sham, cTBS, or iTBS over the left M1. To reduce the number of animals needed, some subjects underwent more than one sham or stimulation protocol; each protocol was separated by an average of 11 months, with a minimum of 4 months (N = 2), and a maximum of 22 months (N = 1). An anatomical T1-weighted image (T_R_ = 7.4 ms_,_ T_E_ = 3.4 ms, FOV = 200 x 200 mm^2^, 170 slices, 1mm^3^ isotropic voxel) was acquired on a Philips Achieva 3.0 T whole-body MRI scanner (Philips Healthcare, Best, The Netherlands), with an eight-channel sensitivity encoding head coil. T1 acquisition allowed for M1 localization and neuronavigation during resting motor threshold measurement and facilitated registration of PET data.

### Resting motor threshold measurement

Resting motor threshold (RMT) was measured in animals under anesthesia before the first TBS or sham session and again 24hr following the last session, usually before a PET scan. The scanning protocol used is standard in our laboratory, has been published extensively [43–45] and is briefly explained in sections below. While the animal was under isoflurane (1–1.5% in 100% oxygen), the left M1 location was identified using their own T1-weighted anatomical MRI and a neuronavigation system (BrainSight). Briefly, EMG electrodes were placed on the right deltoid muscle, and single TMS pulses were delivered using a 70 mm figure-eight coil positioned over the left primary motor cortex. The left M1 ’hotspot’ location was identified and marked with a water-resistant marker to facilitate coil placement during TBS delivery.

We measured RMT as the lowest % of maximum stimulator output (MSO) to produce motor evoked potentials ≥ 50 μV in the relaxed deltoid muscle in five out of 10 consecutive trials [46]. Animals remained under light anesthesia during M1 localization and RMT measurement.

### TBS delivery

Chronic TBS or sham stimulation delivery started the day following RMT measurement. Stimulation was administrated on the awake animal Monday to Friday over the left M1. The number of sessions was adjusted based on PET scan and tracer availability (average iTBS = 17 ± 3; average cTBS sessions = 16 ± 3; average sham sessions = 16 ± 2). One animal underwent 22 iTBS stimulations due to a breakdown of the PET scanner that pushed the scans back by a week.

TBS parameters were chosen to match parameters from clinical studies. We delivered a total of 600 pulses in bursts of three stimuli at 50 Hz, at a frequency of 5 Hz over the left M1 [47]. Stimulation was administrated using a Magstim Rapid^2^ stimulator with a D70^2^ figure-of-eight coil (Magstim Co., UK) at 90% RMT. Continuous TBS was applied for 40 seconds in a continuous train, while iTBS was delivered for 190 seconds in 10-second intervals, consisting of 2-second trains of TBS followed by 8 seconds of no stimulation. Sham stimulation was administered following cTBS parameters but orienting the coil "upside-down" (i.e., magnetic field facing away from the head of the subject with the coil at least 7–8 cm from the skull) and at an intensity of 15% MSO.

During stimulation, the animal sat quietly in a primate chair, restrained only by the collar. All animals were habituated to regular handling, but as a precaution, they received a mild tranquilizer (low dose of a ketamine/xylazine mixture, 7:3, 0.1 to 0.2 mL of the mixture IM) during the first couple of sessions, as they got accustomed to TBS application. While gently stabilizing the head of the animal with one hand, TBS was delivered over the skin-marked M1 location on the left hemisphere. After the end of TBS stimulation, the monkey sat quietly for at least 5 minutes and was then walked back and released into its home cage. No abnormal behavior was noticed in any animals after TBS or sham stimulation.

### Positron emission tomography

PET studies were performed on a Siemens High-Resolution Research Tomograph (ECAT HRRT, CPS Innovations, Knoxville, TN, USA) with a 3-dimensional resolution of 2.5 mm at the University of British Columbia. Synthesis of [^11^C]PBR28 is explained elsewhere [48]. Two [^11^C]PBR28 scans were acquired: one before (baseline) and one 24 h after the last chronic stimulation delivery. In total, evaluations were carried out in 7 animals that received cTBS, 8 animals that received iTBS, and 7 animals that received sham stimulation.

**Fig 1:**
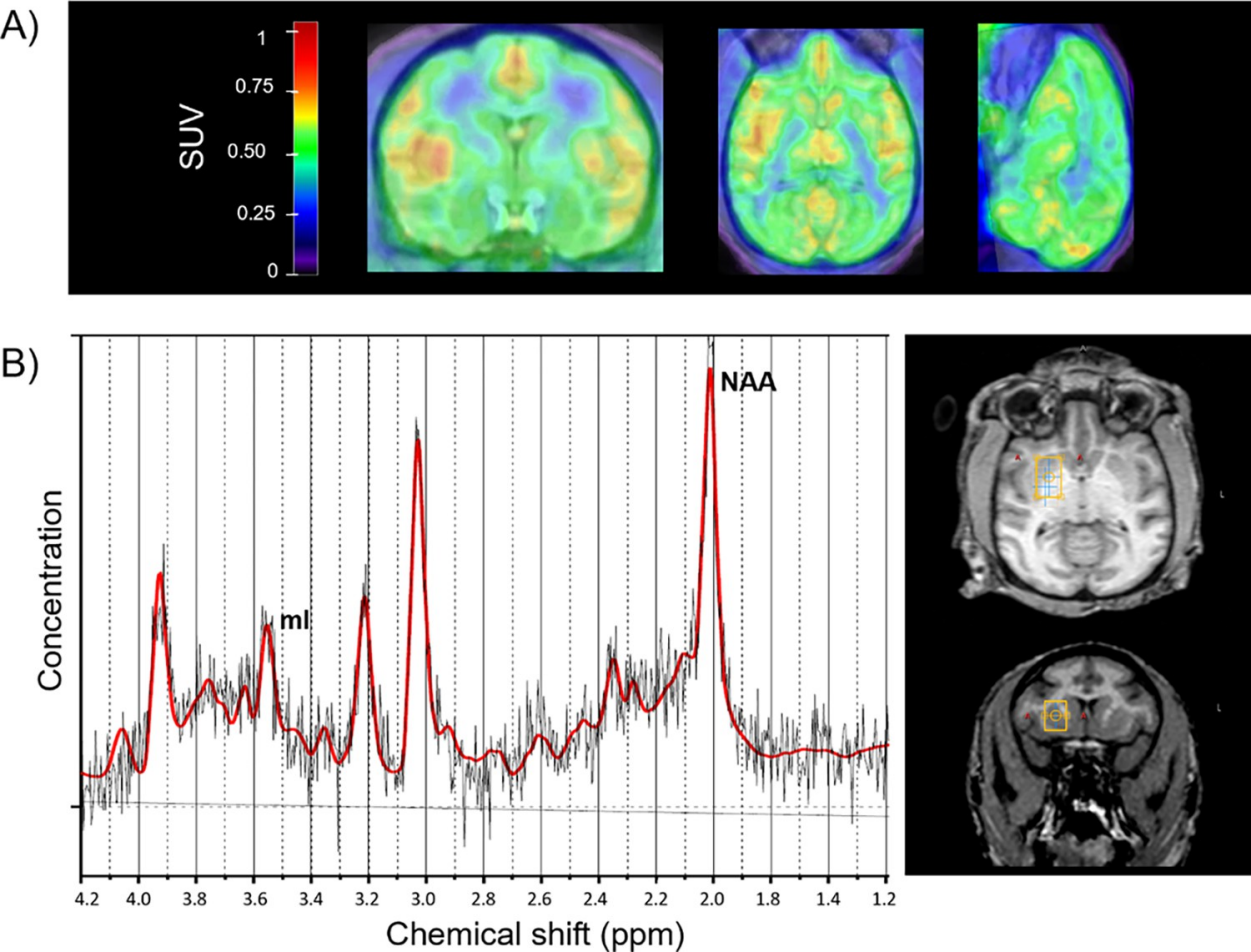
PET and MRS experiments. A) PBR28-PET image overlayed on T1 template. B) Voxel location on the right hemisphere and representative MRS spectra.

The procedure for [^11^C]PBR28 scan acquisition in NHP has been previously described [43]. Briefly, the animal was sedated (ketamine, 10 mg/kg IM) and given atropine (0.05 mg/kg IM) to reduce secretions. The NHPs were intubated to administer isoflurane in 100% oxygen. A line was placed in the saphenous vein for PET tracer administration. Monkeys were positioned prone in a custom-made PET-compatible head holder to prevent head movement during scan acquisition. Light anesthesia (isoflurane 1–1.5% in 100% oxygen) was maintained for the duration of the scan. The tracer was delivered in 10 mL saline through IV administration for over 1 minute. Dynamic image acquisition started at tracer injection and ended 90 minutes later. Upon scan completion, anesthesia delivery was terminated. Animals awoke spontaneously and no side effects were noted. Transmission scans were acquired for attenuation correction over ten minutes with a rotating ^137^Cs source. Reconstruction was carried using the 3D list-mode ordinary Poisson Ordered Subset Expectation Maximization (OP-OSEM) algorithm with 16 subsets and six iterations, with corrections for decay, dead-time, normalization, attenuation, scattered and random coincidences. After reconstruction, the images were smoothed with a 2.0-mm full-width at half maximum (FWHM) Gaussian filter to reduce noise. Images were binned into 17 time frames: 5 × 1 min, 2 × 2.5 min, 4 × 5 min, and 6 × 10 min. The frames were spatially realigned for each subject with rigid-body transformation. PET images were registered to an in-house MRI T1 rhesus monkey template (Fig 1) with associated predefined hand-drawn regions of interest: left and right M1, putamen, cerebellum, and white matter of the centrum semiovale.

We calculated the total volume of distribution, using a population based input function (V_T_^PBIF^), and the distribution volume ratio (DVR) to quantify [^11^C]PBR28 in each region of interest. Two DVR estimates were computed, one using the cerebellum and the other using the centrum semiovale (DVR_cereb,_ DVR_WM_) as pseudo-reference regions [49]; data between 20 to 70 minutes post-injection were fitted. V_T_^PBIF^ was estimated using an in-house population-based input function as previously described [43,50]; data between 20 to 70 minutes post-injection were fitted without blood volume correction and no time shift for the input function.

### Magnetic resonance spectroscopy

MRS acquisition was performed 24 to 48 hours before the first stimulation session and 24 hours after the last session using on a Philips Achieva 3.0 T whole-body MRI scanner (Philips Healthcare, Best, The Netherlands), with an eight-channel sensitivity encoding head coil.

MRS spectra were acquired in 9 animals that received cTBS, 7 that received iTBS, and 6 that received sham stimulation. Animals were anesthetized with ketamine (10 mg/kg IM) to allow for preparation and transportation to the MRI suite. During scan acquisition, we delivered low doses of sodium pentobarbital IV (30 mg/kg/h IV) to maintain anesthesia.

A single-voxel 1H-MRS PRESS spectra was acquired (TR = 4000 ms, TE = 30 ms, sampling frequency = 2000 Hz, signal averages = 128, voxel dimensions = 15 mm × 8 mm ×10 mm, water suppression = VAPOR, shimming = Philips Pencil-Beam projection-based method, motion correction = frequency stabilization). The voxel was manually placed over the left and right putamen (Fig 1). LCModel [51] was used to estimate the absolute metabolite concentration of NAA and mI using the reference area of the unsuppressed water signal. The quality of spectral fits was inspected visually to ensure goodness-of-fit and a narrow full width at half maximum. Metabolite concentrations were rejected based on the Cramér-Rao lower bound (CRLB) estimations from LCModel; metabolites were rejected when the CRLB was greater than 25% of the median value of the metabolite concentrations[52]. Two metabolite concentration estimates required rejection on this basis.

Metabolite concentrations were adjusted for the water relaxation of the different tissues within the MRS voxel. First, we used FMRIB’s FAST on T1 images to segment images into three tissue classes: gray matter, white matter, and CSF. MRS voxels were linearly registered into the T1 space and used to calculate the volume of each tissue within the voxel. Finally, tissue volume fractions were used to correct NAA and mI concentration estimates for water relaxation using values from the literature [53,54].

### Post-mortem evaluation

After completing the PET studies, subjects received and additional stimulation session of TBS or sham–based on the paradigm that was just delivered–and were euthanized 24 h later using a previously described method [2]. The NHPs were anesthetized with ketamine (10 mg/kg IM), taken out of their cage and immediately injected with sodium pentobarbital in a saphenous vein (Euthanol; 120 mg/kg IV). They were then perfused transcardially with isotonic saline, followed by 3–4 L of freshly prepared 4% paraformaldehyde. The brain was dissected and placed whole in a jar of cold 4% paraformaldehyde for another 48–72 h at 4°C, then switched to a solution of fresh cold PBS that was changed 3 times every couple of days before shipment to the laboratory of Dr. Martin Parent at Laval University. Brains were shipped with FedEx overnight.

In total, 3 animals were euthanized after sham stimulation, 3 after iTBS, and 4 after cTBS. To minimize the effect of active TBS in our sham cohort, one of the subjects received only sham stimulation (i.e., underwent only one stimulation cycle). For the remaining two subjects, there was a time of 5 and 8 months between active TBS and sham stimulation to prevent “carry-over” effects.

Brains were cut with a vibratome (VY1200 S’ Leica) into 50 μm-thick transverse sections, and sections were selected from each NHP brain at the anteroposterior (AP) coordinate of -7 mm relative to the anterior commissure (Bowden and Martin, 2000). These were incubated in a blocking solution made of 0.1% Triton X-100, and 2% normal donkey serum (NDS) diluted in phosphate buffer saline (PBS) (0.1M, pH 7.4). Primary antibody incubation was made overnight at 4°C using anti-TSPO made in goat (1:200, product no. AP31284PU-N, Origene) and anti-TMEM119 (1:200, product no. ab209064, Abcam). Secondary antibody incubation lasted 2 h at room temperature using FITC anti-goat made in donkey (1:400, product no. 705-095-147, Jackson) and 594 anti-rabbit made in donkey (1:400, product no. 711-585-152, Jackson). All antibodies were diluted in the blocking solution. Finally, incubation with DAPI (100 ng/mL, product no. D-1388, Sigma) was made for 10 minutes for soma identification. Sections were then mounted on gelatin-coated slides and coverslipped using Dako fluorescence mounting medium (product no. S3023, Dako).

**Fig 2:**
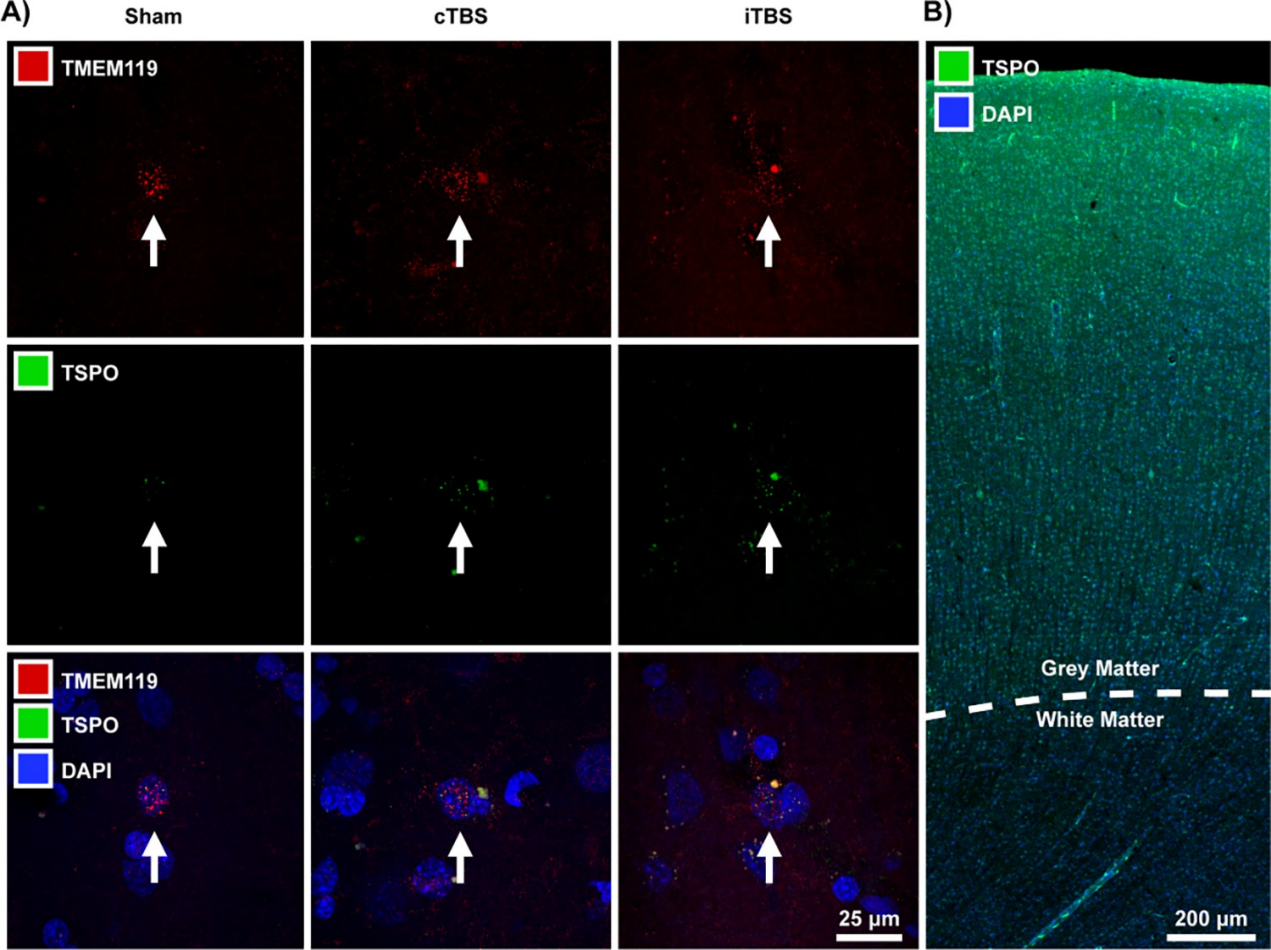
Post mortem staining. (A) Images of fluorescent staining for TSPO, TMEM119 and DAPI across the three conditions. Within each condition, split images show individual fluorescent staining for TSPO and TMEM119 with the third image showing the combined staining in addition to DAPI. The arrows point to a single microglia within each condition as identified using TMEM119. (B) Low magnification image of the fluorescent staining for TSPO and DAPI in the M1 and centrum semiovale.

Stained sections were visualized with a confocal microscope (LSM700, Zeiss). Fluorescent staining for TMEM119, a protein expressed exclusively in microglia, was used to identify microglia and confirm that TSPO localization within these cells (Fig 2). Then by using the Zen software, z-stacks of 35 μm were acquired with a 10x objective from the regions of M1, putamen, and centrum semiovale. These image stacks were then processed using maximum intensity projection, and the mean fluorescence intensity of TSPO staining was recorded from the software. An image of the fluorescent staining is shown in Fig 2. Mean fluorescence intensity from M1 and putamen was reported as a ratio to the mean fluorescence intensity obtained in the centrum semiovale (%WM).

### Statistical analysis

Paired t-tests were used to evaluate differences between baseline and post-stimulation estimation of PET and MRS measurements. Correction for multiple comparisons used the Holm-Sidak method. Unpaired t-tests were used to evaluate the difference between the sham and TBS cohorts from the post-mortem data. All statistical analyses were conducted using GraphPad Prism version 9.0.0 for Windows.

## Results

### PET-TSPO quantification

We used [^11^C]PBR28 to evaluate TSPO in vivo before and after sham stimulation (n = 7), cTBS (n = 7) and iTBS (n = 8). There was no significant difference between regions of the left and right hemisphere in both baseline and post-stimulation conditions. Paired t-test showed no significant differences between baseline and post-stimulation V_T_^PBIF^ after sham (p > 0.6), iTBS (p > 0.9), or cTBS (p > 0.7) in left and right M1. Similarly, there was no significant changes post intervention in the putamen (p > 0.4, see Table 1).

**Table 1:** Summary of total volume of distribution computed with a population-based input function.

	Sham			iTBS			cTBS		
	Avg Baseline	Avg Post	% Diff (p val)	Avg Baseline	Avg Post	% Diff (p val)	Avg Baseline	Avg Post	% Diff (p val)
**M1**									
L	43.8	33.4	-23	39.3	32.5	-17	38.4	45.1	17
	± 20	±10	(0.7)	± 15	±9	(0.7)	± 19	±12	(0.6)
R	41.7	31.4	-24	39.2	36.4	-7	38.4	43.3	13
	± 18	±9	(0.6)	± 15	±16	(0.8)	± 20	±11	(0.6)
**Putamen**									
L	52.2	42.7	-18	50.9	44.2	-13	47.1	51.8	10
	± 25	±13	(0.7)	± 22	±18	(0.8)	± 23	±13	(0.6)
R	54.7	41.3	-24	51.3	41.5	-19	47.2	54	14
	± 28	±12	(0.7)	± 22	±15	(0.7)	± 24	±12	(0.6)

Total Volume of Distribution (V_T_^PBIF^) before (Baseline) and after (Post) theta burst stimulation (TBS). Values are presented as the average across subjects ± SD and the average percentage difference (Diff) between the two time points. Corrected p values from paired t-test are also shown.

Tracer quantification was also carried with DVR using the cerebellum as a reference region (DVR_cereb_) [49]. Similar to V_T_^PBIF^ values, paired t-test revealed no significant differences between baseline and post-stimulation DVR_cereb_ estimates in M1 and putamen after chronic delivery of sham stimulation (p > 0.2), iTBS (p > 0.9), and cTBS (p > 0.9) (see Table 2). DVR was also computed using WM as a reference region, specifically WM from the centrum semoviovale (DVR_WM_), to facilitate comparison of PET and post-mortem evaluations (see below). DVR_WM_ estimates did not significantly change in M1 and after chronic sham (p > 0.9), iTBS (p > 0.9), and cTBS (p > 0.08) (Table 2).

**Table 2:** Summary of distribution volume ratio calculated using cerebellum (DVR_cereb_) and white matter (DVR_WM_) as reference regions.

	Sham				iTBS				cTBS		
	Avg Baseline	Avg Post	% Diff (p val)		Avg Baseline	Avg Post	%Diff (p val)		Avg Baseline	Avg Post	% Diff (p val)
**DVR**_**cereb**_											
**M1**											
L	1.01	0.97	-4		1.0 3	1.05	2		0.96	0.97	1
	±0.2	±0.07	(0.9)		±0.1	±0.2	(0.9)		±0.1	±0.09	(0.9)
R	0.98	0.94	-4		1.02	1.05	3		0.95	0.95	0
	±0.2	±0.08	(0.9)		±0.1	±0.2	(0.9)		±0.1	±0.08	(0.9)
**Putamen**											
L	1.21	1.27	5		1.35	1.34	-1		1.19	1.22	3
	±0.2	±0.06	(0.8)		±0.2	±0.2	(0.9)		±0.1	±0.08	(0.9)
R	1.18	1.26	6		1.35	1.32	-2		1.2	1.23	3
	±0.1	±0.1	(0.2)		±0.2	±0.2	(0.9)		±0.09	±0.08	(0.9)
**DVR**_**WM**_											
**M1**											
L	1.47	1.46	-1		1.5	1.49	-1		1.43	1.62	14
	±0.1	±0.2	(0.9)		±0.1	±0.2	(0.9)		±0.1	±0.1	(0.1)
R	1.44	1.43	-1		1.48	1.5	1		1.41	1.57	12
	±0.2	±0.2	(0.9)		±0.1	±0.2	(0.9)		±0.1	±0.2	(0.1)
**Putamen**											
L	1.89	1.9	0.5		1.97	1.91	-3		1.83	2	9
	±0.2	±0.3	(0.9)		±0.2	±0.3	(0.9)		±0.3	±0.2	(0.1)
R	1.79	1.9	6		1.98	1.92	-3		1.82	2.04	12
	±0.3	±0.2	(0.9)		±0.2	±0.4	(0.9)		±0.3	±0.1	(0.1)

Baseline and post-theta burst stimulation (TBS) values are presented as the average across subjects ± SD, and the average percentage difference (Diff) between the two time points. Corrected p values from paired t-test are also shown.

### NAA and mI concentrations

We carried MRS assessments before and after sham delivery (n = 6), cTBS (n = 9) and iTBS (n = 7) to evaluate concentrations of NAA and mI in the putamen (Fig 1), a region with dense anatomic connections o M1 [55] (Table 3).

**Table 3:** Metabolite concertation corrected for tissue volume.

	NAA								Myoinositol							
	Concentration				%SD				Concentration				%SD			
	Base	Sham	cTBS	iTBS	Base	Sham	cTBS	iTBS	Base	Sham	cTBS	iTBS	Base	Sham	cTBS	iTBS
L	6.45	5.91	6.34	6.21	8%	9%	7%	8%	3.85	4.89	4.94	5.93	10%	9%	6%	7%
	4.59	6	–	–	21%	9%	–	–	6.68	4.2	–	–	11%	10%	–	–
	7.64	8.11	–	8.65	5%	5%	–	5%	6.83	6.64	–	6.13	6%	6%	–	7%
	6.98	7.05	5.68	6.62	6%	7%	11%	9%	6.38	7.06	5.85	6.94	7%	6%	9%	7%
	8.04	8.33	7.67	–	6%	7%	6%	–	5.83	6.15	6.32	–	8%	7%	6%	–
	8.13	7.82	8.2	7.45	5%	5%	5%	5%	5.56	5.18	4.74	5.34	7%	7%	8%	7%
	6.58	–	7.02	–	6%	–	5%	–	7.21	–	4.99	–	6%	–	6%	–
	8.94	–	8.44	8.17	4%	–	4%	5%	6.05	–	6.34	6.05	6%	–	6%	7%
	6.3	–	7.39	–	7%	–	6%	–	5.88	–	6.59	–	7%	–	7%	–
	7.71	–	7.45	8.54	5%	–	8%	5%	5.96	–	6.92	5.65	7%	–	7%	7%
	8.41	–	8.2	8.38	5%	–	5%	4%	4.96	–	4.74	4.85	8%	–	8%	9%
R	6.87	7.24	7.77	6.61	6%	6%	5%	7%	5.54	6.09	5.93	6.56	7%	7%	6%	6%
	6.61	5.92	–	–	8%	9%	–	–	5.57	5.4	–	–	8%	8%	–	–
	7.36	7.58	–	7.56	6%	6%	–	6%	6.2	6.43	–	6.07	7%	7%	–	7%
	8.48	6.38	7.34	7.18	5%	7%	6%	7%	4.99	5.52	6.29	6.26	10%	8%	7%	7%
	6.85	7.39	7.49	–	6%	6%	6%	–	6.09	6.32	5.31	–	6%	6%	7%	–
	**	7.67	7.65	8.2	**	5%	5%	5%	**	5.66	4.77	5.23	**	7%	8%	7%
	7.67	–	8.86	–	7%	–	5%	–	7.27	–	7.35	–	7%	–	6%	–
	8.86	–	8.36	8.84	4%	–	4%	5%	6.03	–	6.37	7.27	6%	–	6%	6%
	6.5	–	7.1	–	6%	–	6%	–	7.05	–	6.16	–	6%	–	7%	–
	7.29	–	8.25	8.22	6%	–	5%	5%	6.14	–	5.45	6.36	7%	–	8%	6%
	8.87	–	8.48	8.27	4%	–	4%	5%	5.36	–	5.06	5.58	7%	–	7%	7%

% SD = estimated standard deviations (Cramer-Rao lower bounds) expressed in percent of the estimated concentrations. ** = Excluded metabolites.

Paired t-test demonstrated there were no significant changes in NAA and mI in the left putamen after sham (NAA: p = 0.7, mI: p = 0.8), iTBS (NAA: p = 0.6, mI: p = 0.7), and cTBS (NAA: p = 0.7, mI: p = 0.9) compared to baseline. Similarly, no significant changes in the right putamen were found after sham (NAA: p = 0.6, mI: p = 0.4), iTBS (NAA: p = 0.8, mI: p = 0.3) and cTBS (NAA: p = 0.5, mI: p = 0.6).

### Post-mortem evaluation

Unpaired t-test showed no significant difference in %WM for TSPO between sham and stimulated animals in the M1 (p > 0.5). Evaluation of %WM in the putamen yielded similar results as there were no significant differences between the sham and cTBS cohort nor between the sham and iTBS (p > 0.2). Individual TSPO %WM are shown in Table 4.

**Table 4:** Normalized mean TSPO fluorescence intensity (%WM).

	Motor Cortex		Putamen	
	Left	Right	Left	Right
Sham	258.2%	220.3%	249.8%	234.9%
	236.6%	245.1%	158.8%	179.7%
	295.1%	191.7%	177.0%	199.8%
cTBS	320.6%	383.5%	385.8%	372.6%
	300.5%	293.2%	209.7%	240.6%
	275.3%	321.6%	240.2%	223.0%
iTBS	231.4%	179.8%	171.3%	146.6%
	390.4%	483.1%	330.1%	304.7%
	283.6%	337.2%	236.7%	203.7%
	259.1%	297.0%	224.9%	279.2%

Mean fluorescent intensity in motor cortex and putamen normalized by mean fluorescent intensity in centrum semiovale.

## Discussion

The current work assessed markers for injury and glial activity following chronic TBS in healthy non-human primates. NAA and mI concentrations, and TSPO expression, were evaluated as markers for neuroinflammatory and injurious processes using PET, MRS, and immunofluorescence imaging.

### Microglia and neuroinflammation

In studies of neuroinflammation *in vivo*, PET-TSPO is a widely used technique. TSPO is thought to be involved in multiple cellular functions such molecular transport, oxidative stress, apoptosis, metabolism, and mitochondrial homeostasis and respiration [56,57]. While its specific physiological functions are still unclear, TSPO is the molecule of choice for PET studies of neuroinflammation as it is over-expressed in activated microglia [21,25,58].

Similar to TSPO overexpression, concentration of mI, an organic osmolyte present in glial cells, has been proposed as a marker for glia, specifically activated astrocytes. Concentration of mI is elevated in an array of pathologies thought to involve neuroinflammation [27,59–61] and has been shown to be a selective glial marker in rat brain tissue [28]. Studies of viral infections and multiple sclerosis have reported a relationship between increase in mI and PET-TSPO uptake [62,63]. Furthermore, MRS evaluation of NAA, a selective marker for mature neurons, allows for the simultaneous assessment of cellular damage that might be concurrent with neuroinflammation. By combining both techniques, PET-TSPO and MRS, we evaluated different components of the neuroinflammatory process.

Three [^11^C]PBR28 measurements were calculated, V_T_^PBIF^_,_ DVR_cereb,_ and DVR_WM._ The latter was computed to improve comparability to immunofluorescence imaging measurements normalized using centrum semiovale estimates. [^11^C]PBR28 measurements showed no significant difference between tracer binding before and after cTBS, iTBS, and sham stimulation. While the V_T_^PBIF^ measurements showed relatively large differences between pre and post values, these differences are within reported test-retest reproducibility ranges in humans (variability ranging from 13.8% to 25.9% [64]) and rhesus monkeys (variability of 23.7% [43]). This lack of significance was consistent across the different measurements and different motor brain regions. MRS evaluation of mI concentration showed similar results, as no significant changes were found after stimulation, and sham delivery in the bilateral putamen.

Correspondingly, immunofluorescence analysis showed that microglial expression of TSPO in the M1 and putamen was comparable between the sham and active TBS cohorts. While limited by the small number of subjects in the post-mortem evaluation, these results, in conjunction with the in vivo assessment, consistently demonstrate no change in glial activation markers, suggesting no evident neuroinflammatory induction after chronic stimulation. Furthermore, the unchanged concentrations of NAA after stimulation suggest there are no changes in neuronal density, which in turn might indicate that stimulation did not induce injurious effects that resulted in the decrease of neurons or neuron viability.

Our findings align with previous studies in healthy rodents, where authors reported no changes in microglia and astrocyte reactivity after 3 and 5 sessions of high and low frequency rTMS stimulation [10,11,65]. In contrast, in rat models of spinal cord injury and chronic pain and depression, rTMS decreased microglia activation and inflammatory markers after 8 and 4 weeks of daily rTMS [66,67]. This discrepancy might suggest that rTMS modulates microglia activity when it is already in an active state and might have no effects when delivered in healthy subjects, as indicated in a recent literature review [65]. Alternatively, discrepancy of results might stem from differences in the study design, such as the subject brain size and stimulated areas (non-human primate versus rodent brain size), or the number of stimulation sessions (3 weeks of stimulation delivered in the current study versus the 4 and 8 weeks of delivery by [66,67]). Thus, further studies should evaluate rTMS effects on neuroinflammatory processes in the diseased brain and assess if rTMS-induced changes are anti-inflammatory, as previously reported [66–68], pro-inflammatory, or if rTMS has no effect over neuroinflammatory processes.

### Microglia and neuroplasticity

Glial cells contribute and regulate synaptic plasticity as they are involved in the development, formation and pruning of neuronal synapses and circuitry [69,70]. A recent study showed rTMS modulation of synaptic plasticity in the presence of modulation of neuroinflammatory markers [71], specifically metalloproteases which are released by activated microglia and astrocytes during neuroinflammatory and neurodegenerative processes. In individuals with mild cognitive impairment, 4 weeks of daily 10Hz chronic rTMS enhanced visuospatial performance, decreased plasma metalloprotease levels, and raised metalloprotease-related tissue inhibitors. Notably, it’s uncertain whether they stem from glia-mediated synaptic plasticity induction or rTMS-induced downregulation of inflammatory processes. Moreover, modulation of synaptic plasticity may involve other metalloprotease-producing cells like endothelial cells and neurons. Thus, further research is needed to determine whether glia serves as one of the mediators of rTMS.

Work from Eichler and colleagues addressed this knowledge gap using in vitro and in vivo approaches [72]. They reported rTMS-mediated release of plasticity-promoting cytokines from resting microglia in organotypic brain tissue cultures. In vivo experiments showed acute 10Hz stimulation increased excitatory postsynaptic potential in control mice while no changes were found in microglia-depleted subjects. These results suggest that glia might be a relevant component of rTMS mediated plasticity modulation, yet validation in human and more relevant animal species is still necessary.

Most studies of rTMS and neuroinflammatory markers are carried out in patient population or animal models characterized by increased neuroinflammation, which makes it difficult to disseminate whether rTMS therapeutic effects are mediated (at least partially) through glia-dependent synaptic plasticity induction, neuroinflammatory modulation or a combination of both.

While our neuroimaging data showed no significant changes in microglia activation markers, we found–in the same subjects–a decrease in cortical excitability in following chronic cTBS, measured as an increased in RMT [2]. However, the lack of changes in microglia activation markers does not necessarily indicate microglia do not contribute to rTMS-induced plasticity. Reports from a electrophysiological study in healthy tissue suggested that resting, rather than active, microglia modulate synaptic activity [73]. Thus, it is possible that microglia contributed to the modulation of synaptic plasticity but, given the nature of the markers evaluated, it was not measurable. Ultimately, the relation between microglia and rTMS-induced plasticity is beyond the reach of the current work, yet it provides relevant information for future study designs aiming to evaluate this interaction.

The current study was designed to mimic clinical TBS delivery, but given the characteristics of our animal subjects, several differences between human and non-human primates should be considered when translating these results. One such difference is the presence of facial muscles inserted at the top of the rhesus monkey’s head that, along with their thick skulls, create a bigger gap between the coil and the stimulated cortex. In addition, a smaller head-to-coil ratio, due to the animal’s head size and the size of the coil we used (a 70mm figure-eight human coil), affects the focality of the delivered stimulation, making the overall delivered stimulation likely less deep and focal than in human studies. Given that coil distance and positioning have been reported as a source of variability of rTMS effects in neuronal activity [74,75], it is unclear whether the same would be true for its effects on glial cells.

## Conclusion

Current work centered on the evaluation of glial activation as an indirect way to evaluate rTMS safety; however, it is worth noting that previous research has also explored the protective effects of stimulation mediated through glial cells (for a comprehensive review, refer to [65]). Nonetheless, the number of available studies in this area remains limited and has predominantly been conducted in rodents, thus constraining its applicability to human subjects. It is still unclear whether glial mediated effects of rTMS are beneficial, detrimental, or limited. To fully understand the therapeutic benefits of rTMS and its safety limitations, it will be critical to understand its effects on the different types of brain cell types, particularly glial cells due to their abundance in the brain.

In the context of safety, the evaluation of microglia and astrocytes provides an added value, as it enables the evaluation of molecular markers of potential adverse effects of stimulation that might otherwise be missed when using conventional metrics such as such as the incidence of headaches, neck pain, and seizures. Our findings revealed that the sustained administration of cTBS and iTBS in the healthy brain does not elicit alterations in markers commonly associated with neuroinflammation (microglia and astrocyte activation) and injuries. Our findings provide novel insight into the safety of TBS and might indicate the absence of neuroinflammatory response, even after multiple daily stimulation sessions at a high frequency in a healthy brain. Future studies should continue to investigate molecular and cellular markers of rTMS safety, in healthy and vulnerable populations, especially when delivered chronically to assure ethical application.
